# Case Report: Secondary Hemophagocytic Lymphohistiocytosis (sHLH) and *Candida auris* Fungemia in Post-acute COVID-19 Syndrome: A Clinical Challenge

**DOI:** 10.3389/fmed.2022.835421

**Published:** 2022-06-17

**Authors:** Sachin Gautam, Gaurav Sharma, Sumeet Singla, Sandeep Garg

**Affiliations:** Department of Internal Medicine, Maulana Azad Medical College and Associated Lok Nayak Hopital, New Delhi, India

**Keywords:** post acute covid-19 syndrome (PACS), secondary hemophagocytic lymphohistiocytosis, *Candida auris*, macrophage activation syndrome (MAS), intravenous immunoglobulin, cytokine storm syndrome (CSS), multisystem inflammatory syndrome-adults (MIS-A), hyperferritinemia syndrome

## Abstract

Severe acute respiratory syndrome coronavirus 2 (SARS-CoV-2) infection causes a disease (COVID-19) with multisystem involvement. The world is now entering a phase of post-COVID-19 manifestations in this pandemic. Secondary hemophagocytic lymphohistiocytosis (sHLH) is a life-threatening hyperinflammatory event triggered by viral infections, including SARS-CoV-2. Both Multisystem Inflammatory Syndrome-Adults (MIS-A) and Cytokine Storm Syndrome (CSS) are considered close differentials of sHLH and add to the spectrum of Post-acute COVID-19 syndrome (PACS). In this report, we presented the case of a middle-aged Asian man who was initially discharged upon recovery from severe COVID-19 infection after 17 days of hospitalization to a private institute and later came to our hospital 13 days post-discharge. Here, he was diagnosed with sHLH, occurring as an extension of CSS, with delayed presentation falling within the spectrum of PACS. The diagnosis of sHLH was made holistically with the HLH-2004 criteria. Our patient initially responded to intravenous immunoglobulin (IVIG) and dexamethasone, later complicated by disseminated *Candida auris* infection and had a fatal outcome. Though many cases of HLH during active COVID-19 and a few cases post COVID-19 recovery have been reported, based on H-score, which has limitations as a diagnostic tool. We report the first case report of post-COVID-19 sHLH using the HLH-2004 criteria, complicated by disseminated Candidemia, emphasizing that the care of patients with COVID-19 does not conclude at the time of hospital discharge. We highlight the importance of surveillance in the post-COVID phase for early detection of sHLH which may predispose to fatal opportunistic infections (OIs).

## Introduction

The pandemic caused by severe acute respiratory syndrome coronavirus 2 (SARS-CoV-2) has overwhelmed healthcare facilities around the world. COVID-19 has an extended spectrum in the post-acute phase with an evolving list of complications. Prieto-Perez et al. first described post-COVID-19 secondary hemophagocytic lymphohistiocytosis (sHLH), indicating a systemic immune-inflammatory disease (1). HLH has been divided into primary and secondary subtypes. Primary HLH is familial, primarily seen in children, whereas sHLH is an adult entity due to underlying infection, autoimmune disease, malignancy, or “post-allogeneic” hematopoietic stem cell transplantation (2). HLH is a life-threatening hyperinflammatory state characterized by multiorgan dysfunction, adding to the spectrum of Post-Acute COVID-19 Syndrome (PACS) (3). Lledo et al. defined the PACS as a clinical entity when symptoms persist beyond four weeks (4). Similarly, the Center for Disease Control (CDC) has formulated “post-Covid conditions” to describe the health issues that persist more than four weeks after being infected with COVID-19 (5). Here, we present the first case report of post-COVID-19 sHLH occurring as an extension of the cytokine storm syndrome (CSS) falling under the timeline of PACS, who initially responded to dexamethasone and intravenous immunoglobulin (IVIG). However, his course was complicated by disseminated Candidemia and he succumbed to his illness. It is prudent to identify this fatal complication early to improve survival.

### Case Report

A 36-year-old farmer, who had been admitted 29 days ago to another hospital with severe COVID-19 pneumonia, presented to our emergency 13 days after discharge with complaints of fever (101.4°F) for 5 days, generalized weakness and loss of appetite for 4 days, progressive exertional dyspnea, and occasional dry cough for 2 days. He had no history of orthopnea, paroxysmal nocturnal dyspnea, pedal edema, hemoptysis, chest pain, burning micturition, or bowel complaints. He had been discharged from the last admission on home-based intermittent oxygen therapy at 2-4 L/min, multivitamins, and oral pirfenidone (an anti-fibrotic agent for post-COVID-19 pulmonary fibrosis) 200 mg three times a day (TDS). He had no history of substance abuse or chronic illness, and his family history was insignificant.

He was conscious, oriented, and febrile (102.8°F/39.3°C). His oxygen saturation was 93% under ambient air, a respiratory rate of 15 breaths per min, blood pressure of 108/76 mmHg, and a pulse rate of 108 beats per min. On systemic examination, hepatomegaly was present 2 cm below the right subcostal margin in the mid-clavicular line and bilateral end-inspiratory fine crepitations were auscultated in the chest. The rest of the systemic examination was normal.

**Figure 1:**
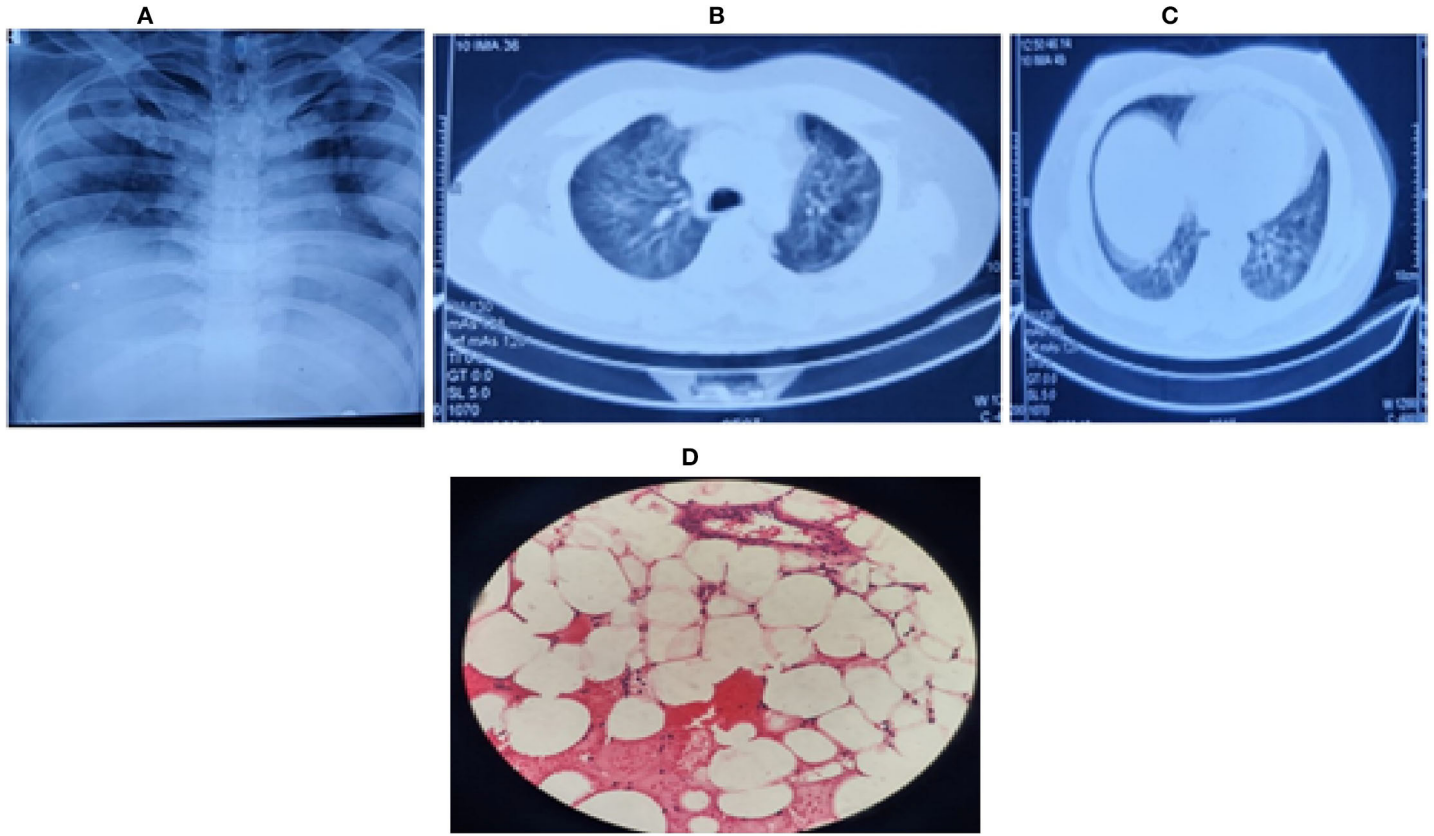
**(A)** Chest radiograph (bedside) on first day, showing bilateral middle zone haziness, **(B)** Contrast enhanced CT (CECT) chest on second day showing bilateral ground-glass opacities in lung parenchyma with **(C)** left upper lobe consolidation and air bronchogram. **(D)** Bone marrow biopsy showing hypocellular marrow (overall cellularity < 15%), lacunar spaces extensively replaced by fat cells; the residual cellularity mostly included lymphocytes, plasma cells, mast cells, and macrophages with only a few hematopoietic cells.

On investigation, his random blood sugar was 203 mg/dL, TruNat swab for SARS-CoV-2 was reported negative, and arterial blood gas analysis showed Type 1 respiratory failure. His ECG showed sinus tachycardia, and the bedside chest radiograph showed bilateral ground-glass opacities (Figure 1A). A provisional diagnosis of post-COVID-19 sequelae with bilateral lower respiratory tract infection (LRTI) with Type 1 respiratory failure was made. Further investigations were sent. He was treated with low flow oxygen support, antipyretics, injectable ceftriaxone, intravenous fluids, and supportive care. His preliminary blood investigations showed pancytopenia with reduced reticulocyte count and transaminitis [Hb-9.2 g/dL, TLC-3,400 cells/mm^3^, platelets-20,000 cells/mm^3^, corrected reticulocyte count < 0.5%, peripheral smear-pancytopenia, serum AST-4688 U/L, ALT-6680 U/L, serum bilirubin 0.8 mg/dL, and ALP-186 U/L]. Based on these, possible differentials were viral infection, post-acute COVID-19, steroid-induced immunosuppression, drug-induced liver injury (DILI) probably due to Pirfenidone, Multisystem Inflammatory Syndrome–Adult (MIS-A), or COVID-19 associated CSS. Biochemical analyses have been tabulated in Table 1. Serum serologies [for HIV 1 and 2, hepatitis A, B, C, D, and E, Epstein-Barr virus (EBV), parvovirus B19, herpes simplex virus (HSV), cytomegalovirus (CMV), dengue, HSV 1 and 2, VDRL, Rickettsia, Leptospira, and scrub typhus], peripheral blood smear for malarial antigens, and serum autoimmune profile (anti-nuclear antibodies *via* immunofluorescence assay) were sent.

**Table 1:** Showing laboratory parameters during the current hospital stay.

**Parameters**	**Day 1**	**Day 3**	**Day 5**	**Day 6**	**Day 7**	**Day 9**	**Day 11**	**Day 13**	**Day 14**
Hb [12–15.5 g/dL]	8.8	8.9	9.1	9.3	10.4	10.8	10.6	10.8	10.7
TLC [4000– 11000 cell per mm^3^]	3,400	3,700	3,840	4,500	5,400	9,800	14,000	13,420	14,700
DLC [60–75% polymorphs, 20–40% lymphocytes, 2–6% monocytes, Eosinophils: 1–4%]	88/10/1/1	77/20/2/1	79/20/1	82/17/1	88/10/1/1	87/12/1	88/11/1	90/8/1/1	91/7/1/1
Hematocrit [For men; 38.3 to 48.6%]	35.9	36.2	35.7	34.7	35.2	35.6			
Reticulocyte count [0.5 to 2.5%.]	0.50	0.7	1.1		1.2				
Platelets 1.5 to 4.0 × 10^9^ cells per mm^3^]	20,000	22,000	66,000	1,25,000	1,55,000	2,50,000	2,65,000	2,34,000	2,13,000
Bilirubin– T/D (1.3 mg/dL)/ 0.3 mg/dL]	0.89	0.48	1.6/1.1	1.2/0.7	1.1/0.9	0.7/0.2	0.6/0.2	0.9/0.2	0.8/0.1
AST [15–45 U/L]	4,688	9,060	11,062	8,500	5,680	1,244	960	546	225
ALT [5–50 U/L]	6,680	9,156	12,100	9,100	6,210	2,380	1,320	939	726
ALP [38–125 U/L]	188	234	218	255	229	178	210	188	33
Blood Urea/ Serum creatinine [(19–43 mg/dL)/(0.66–1.26 mg/dL)]	30/0.5	32/0.6	38/0.9	33/0.8	29/0.7	33/0.8	76/1.8	98/2.8	
Sodium/Potassium [(137–145 mmol/L) /(3.5–5.1 mmol/L)]	137/4.1	133/4.6	140/4.5	137/4.1	141/4.4	143/4.4	138/4.1	142/4.4	143/4.7
Total protein/ Serum albumin [(6.3–8.2 g/dL) / (3.5–5.0 g/dL)]	5.0/2.6	4.8/2.6	4.9/2.7	5.1/2.8	5.0/2.8	5.2/2.9	5.3/2.9		
Uric acid [3.4–7.0 mg/dL (male)]	1.9								
CPK–T/MB [10–120 IU/ L / 5 – 25 IU/L ]		113/56	145/43	125/34			114/36	90/33	113/39
Blood Culture		Sterile	Sterile	Sterile	Sterile		Sterile	*Candida auris* sensitive to caspofungin resistant to fluconazole	*Candida auris* sensitive to caspofungin, resistant to fluconazole
Urine culture		*E. coli* sensitive to nitrofurantoin, colistin, rest all resistant	Sterile	Sterile	Sterile		Sterile	Sterile	Sterile
Urine routine microscopy	No active sediments, no sugar/protein.	No active sediments, no sugar/protein.	No active sediments, no sugar/protein.	No active sediments, no sugar/protein.	No active sediments, no sugar/protein.		No active sediments, no sugar/protein.	No active sediments, no sugar/protein.	No active sediments, no sugar/protein.
Peripheral blood Smear	Pancytopenia with normocytic normochromic blood picture, occasional tear drop cells and elliptocytes seen along with reduced WBCs and platelets.	Bicytopenia with mild anisocytosis, occasional elliptocytes, tear drop cells and few target cells seen. No atypical cells or schistocytes seen.	Bicytopenia with reduced platelet counts, shift to the left in WBCs, toxic granules in neutrophils seen. No atypical, haemoparasites or schistocytes seen.	Thrombocytopenia with large sized platelets seen.					
**Serum Inflammatory Markers**									
LDH [120–146 U/L]	8,918	9,340	14,000	12,000	9,800	8,700	11,300	13,400	13,788
D–dimer [ <500 ng/mL]	>5,000	>5,000	>5,000	4,700	3,600	3,320	>5,000	>5,000	>5,000
INR [0.9 to 1.1]	1.3	1.6	1.4	1.2	1	1	1.1	1.1	1
Hs–CRP [0.0–5.0 mg/L]	13.11	7.38	45	66	52	44	76	112	156
IL−6 [<7 pg/mL]	2.8	5.6	19.8	24	20	21	76	123	233
NT–PRO BNP [< 125 pg/mL for age 0–74 years]	67	72	109	78	67	60	187	118	104
Serum Ferritin [Male = 30–400 ng/mL]	>2,000	3,900	4,360	1,580	1,270	1,200	>2,000	>2,000	>2,000
Serum Procalcitonin [Low risk: <0.5, high risk: > 2 ng/mL]	0.9	2.5	3.2	2.9	1.7	1.2	2.9	3.3	4.7
ESR [0 to 22 mm/h in 1^st^ h for men]	23			18					
Fibrinogen [200 to 400 mg/dL]		128	119	132	178	186	218	208	213
**Metabolic profile**									
HbA1C [%]	7.4								
Total cholesterol [0–200 mg/dL]		198							
Triglyceride [0–150 mg/dL]		750	950	877	764	721	796	810	877
LDL [40–60 mg/dL]		47							
HDL [< 40 mg/dL]		38							

Further investigations revealed an elevated serum lactate dehydrogenase (8918 U/L), serum D-dimer (> 5,000 ng/mL), serum ferritin (> 2,000 ng/ml), INR (1.6), and serum fibrinogen levels (128 mg/mL), indicating disseminated intravascular coagulation (DIC) - like picture with a probably viral, drug-induced (pirfenidone, steroids), or autoimmune etiology. Antimicrobials were changed empirically to intravenous piperacillin-tazobactam (4.5 g QID) and doxycycline (100 mg BD) with ursodeoxycholic acid (300 mg TDS). In light of persistent pancytopenia, a bone marrow biopsy was performed under cover of platelets transfusion. Ultrasonography of the whole abdomen revealed hepatomegaly.

On the second day, his dyspnoea worsened, and because of raised D-dimer, a CT pulmonary angiography of the chest was done to rule out post-COVID-19 pulmonary embolism. However, it showed only a consolidation in the left upper lobe and bilateral ground-glass opacities with no evidence of pulmonary embolism (Figures 1B,C). Furthermore, his troponin I and T were negative, with normal creatine phosphokinase (CPK) and N terminal pro-B-type natriuretic peptide (NT Pro-BNP) levels. A transthoracic 2D-ECHO was reported normal. On the third day, serum procalcitonin was raised (2.5 ng/mL), but the blood culture was sterile, and urine culture after 48 h of incubation showed *Escherichia coli* susceptible to colistin which was initiated. Persistent fever prompted us to send repeated blood cultures, fungal cultures, urine routine microscopy, and cultures for the next 3 days. Sputum induction failed as the patient had a dry cough. Meanwhile, all viral serologies were reported negative. The patient persistently had transaminitis [AST-11,000 U/L; ALT-12000 U/L; INR-1.6 (refer to Table 1, day-5)], with no bleeding manifestations or altered sensorium throughout his hospital stay. Empirically vitamin K, rifaximin, and lactulose were added to the treatment. Meanwhile, his serum beta-D glucan level, galactomannan level, and autoimmune profile, including anti-nuclear antibodies (ANA), p-ANCA, c-ANCA, and thyroid profile, were reported normal.

After correlating pancytopenia, hepatomegaly, and DIC, with a consistently raised serum ferritin (> 4,000 ng/ml), serum triglycerides (950 mg/dL) and reduced serum fibrinogen levels (< 150 mg/dL) with an ESR of 23 mm (during the first hour) with his clinical presentation, we contemplated the possibility of secondary HLH (Hemophagocytic lymphohistiocytosis) as part of CSS due to COVID-19 infection. Meanwhile, bone marrow biopsy showed a hypocellular marrow for his age (overall cellularity < 15%), with a possibility of aplastic anemia, malignancy, or an invasive pathology (Figure 1D). After correlating the clinical, biochemical, radiological, and bone marrow reports, we diagnosed sHLH based on the HLH 2004 criteria and an H-Score of 256 (Table 2A. Our patient fulfilled six of the eight HLH-2004 criteria points with an H-score of 256, indicating a 99% probability of having HLH (Table 2A).

**Table 2A:** Calculation of H- Score (6) and Center for Disease Control (CDC) Multisystem Inflammatory Syndrome-Adults (MIS-A) criteria (7). H-Score calculation as per the criteria by Fardet et al. (6) An H-Score of 256 was found in our patient, giving us a 99% probability of hemophagocytic lymphohistiocytosis (HLH).

**Serial no**	**Parameters**	**Scoring (CUT OFF) used in H-Score calculation (6)**	**Our patient profile (score)**	**MIS-A Criterion (as per CDC) (7)**
1	Known underlying immunosuppression	0 (no) or 18 (yes)	Yes (18)	• **1. Clinical Criteria:** Documented fever (≥38.0 C) for ≥24 h prior to or within the first THREE days of hospitalization along with atleast THREE of the following clinical criteria including one primary clinical criterion.
2	Temperature (°C)	0 (<38.4), 33 (38.4–39.4), or 49 (>39.4)	39.3°C (33)	• **1A: Primary clinical criteria** • 1.Severe cardiac illness Includes myocarditis, pericarditis, coronary artery dilatation/aneurysm, or new-onset right or left ventricular dysfunction (LVEF <50%), 2nd/3rd degree A-V block, or ventricular tachycardia. • 2.Rash AND non-purulent conjunctivitis.
3	Organomegaly	0 (no), 23 (hepatomegaly or splenomegaly), or 38 (hepatomegaly and splenomegaly)	Hepatomegaly (23)	• **1B: Secondary clinical criteria** • 1.New-onset neurologic signs and symptoms include encephalopathy in a patient without prior cognitive impairment, seizures, meningeal signs, or peripheral neuropathy.
4.	Number of cytopenias	0 (1 lineage), 24 (2 lineages), or 34 (3 lineages)	3 lineages (34)	• 2.Shock or hypotension not attributable to medical therapy. • 3.Abdominal pain, vomiting, or diarrhea • 4. Thrombocytopenia (platelet count < 150,000/ microliter)^*^
5.	Ferritin (ng/mL)	0 (<2,000), 35 (2,000–6,000), or 50 (>6,000)	4360 ng/ml (35)	• **2. Laboratory evidence** • **2A**. Elevated levels of at least TWO of the following: C-reactive protein, ferritin, IL-6, erythrocyte sedimentation rate, procalcitonin^*^
6.	Triglyceride (mmol/L)	0 (<1.5), 44 (1.5–4), or 64 (>4)	950 mg/dL = 10.7 mmol/L (64)	• **2B**. A positive SARS-CoV-2 test for current or recent infection by RT-PCR, serology, or antigen detection^*^
7.	Fibrinogen (gm/L)	0 (>2.5) or 30 (≤ 2.5)	≤ 250 mg/dL = ≤ 2.5 g/L (30)	
8.	Serum Aspartate transaminase (IU/L)	0 (<30) or 19 (≥30)	11062 U/L (19)	
9.	Hemophagocytosis features on bone marrow aspirate	0 (no) or 35 (yes)	No (0)	
10.		Total	256 points, giving us a 99% probability of HLH. (Cut off is 169 points)	^*^Our patient fulfilled (bold and italicized) 1. One secondary clinical criterion– thrombocytopenia. 2. Two laboratory criteria. But sepsis could not be excluded as defined by CDC for MIS-A in our patient since he developed urinary tract infection.

The following etiologies for sHLH were considered: a post-COVID-19 infection, DILI due to pirfenidone, or sepsis-induced HLH (compounded by steroid-induced immunosuppresion). The close differentials were CSS and MIS-A. Nevertheless, autoimmune disease, tuberculosis, and other viral etiologies had been ruled out. Hence, as per HLH treatment protocol, intravenous injection of 10 mg dexamethasone two times a day with 30 g IVIG per day was started.

Furthermore, serum soluble interleukin-2 receptor (sCD25) levels and flow cytometry for immunodeficiency profile were sent. From day six to ten, our patient responded clinically to this treatment. Also, no fever was documented, and no new lung crepitations were heard on auscultation. Moreover, improvements in the biochemical profile and inflammatory markers were seen. Repeated blood, fungal, and urine cultures were reported to be sterile (Table 1, from day 6 to 9).

Later, his sCD25 levels were reported positive, and serum flow cytometry showed a reduction in the percentages of T cells (CD3: 9.21% vs. 53.2% control), B cells (CD19: 1.32% vs. 7.19% control), CD20 cells (1.06% vs. 7.29%), NK cells (CD 56: 0.13% vs 13.54% control), and CD15 (0.26% vs. 11.71% control), along with a reduced activity of NK cells, and yet the CD4:CD8 ratio was maintained. This report suggested humoral and cell-mediated immunosuppression in our patient along with low NK cell activity, which was consistent with the diagnosis of sHLH.

**Figure 2:**
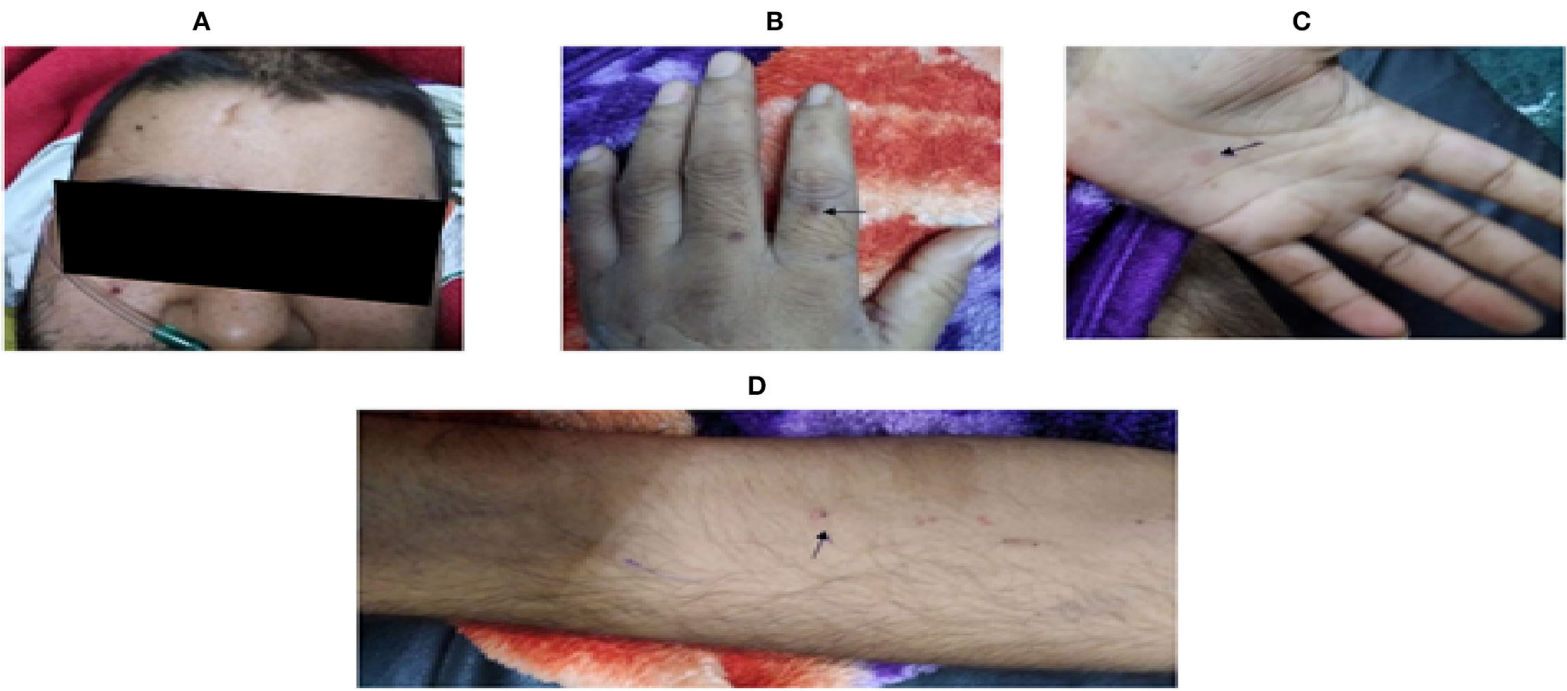
Showing skin lesions observed on eleventh day of hospital stay. (Arrow): Multiple, erythematous, and non-palpable maculopapular lesions over **(A)** face, **(B,C)** palm and dorsum of both hands, and **(D)** flexor aspect of arms.

On the eleventh day, an erythematous, maculopapular rash developed, along with fever (101.7°F) episodes. It was a non-palpable rash measuring 0.5 to 1 cm, on the flexor and extensor aspects of both arms, hands, face, and upper abdomen, suggesting a non-vasculitic but infective etiology (Figure 2). Hence, intravenous teicoplanin was added to cover methicillin- and vancomycin-resistant *Staphylococcus aureus* (MRSA and VRSA) along with the antifungal fluconazole. He also had raised inflammatory markers [serum procalcitonin, lactate dehydrogenase (LDH), D-dimer, C-reactive protein (CRP), and interleukin 6 (IL-6)] with deranged kidney functions tests (refer to Table 1), which raised the possibility of sepsis due to a new hospital-acquired infection in an immunosuppressed state. Blood culture on day thirteen grew *Candida auris*, sensitive to caspofungin. Hence, fluconazole was replaced with caspofungin. His fever subsided, but on day fourteen, his kidney function tests further worsened, and he developed respiratory distress with worsening hypoxemic respiratory failure. His ECG and cardiac biomarkers were normal with a negative TruNat for SARS-CoV-2. A CT angiography of the chest was planned to rule out pulmonary embolism, as inflammatory markers along with d-dimer were raised, but the patient had to be intubated because of worsening respiratory distress. His repeat blood culture on day fourteen was again positive for *Candida auris*, suggesting disseminated Candidemia. He finally succumbed to his illness on day fifteen of hospitalization, despite our best efforts.

The final diagnosis in this case was post-COVID-19 sequelae with superadded pneumonia and sepsis leading to secondary HLH with opportunistic disseminated candidiasis, causing multi-organ failure [acute kidney injury (AKI) with acute respiratory distress syndrome (ARDS)]. Written consent was taken from the patient's relative for using clinical data for this case report.

## Discussion

Cytokine storm syndrome presents as multiorgan failure, rising inflammatory markers, and sHLH (20). CSS can be triggered by any infectious or rheumatological disease and cancer chemotherapy (20, 21). In the respiratory epithelial lining SARS-CoV-2 stimulates the proliferation of CD8+ T-cells and NK cells with cytokines converting histiocytes into activated macrophages that further release cytokines like IL-1, IL-6, and tumor necrosis factor-α, which stimulates CD8+ T-cells proliferation, maintaining the vicious cascade of cytokine storm (22). Similar to CSS, severe COVID-19 infection has also been associated with a dysregulated immune response. It leads to a hyperinflammatory state with elevation of IL-6, IL-8, IL1β, and ferritin (23). A study showed serum levels of IL-18, IFNγ, sFasL, and serum ICAM-1 were significantly elevated among patients with COVID-19 compared to those with classical HLH or MAS, which could guide to differentiate patients with SARS-CoV-2 induced immunosuppression (24). Our patient was previously admitted for severe COVID-19 infection and presented to us with features of sHLH, which could be attributed to an extension of CSS associated with COVID-19 due to a hyperinflammatory state.

Multisystem inflammatory syndrome is commonly seen among the pediatric population after COVID-19 infection (25). However, the CDC (7) has defined a criterion to diagnose MIS-A in adults as shown in Table 2A. MIS-A presents as a post-infectious syndrome with an antibody-mediated immune dysfunction and a varied clinical presentation, but the exact pathophysiology is unknown. (7, 25). The criteria include patients of age ≥ 21 years hospitalized for ≥ 24 hours or with an illness resulting in death with the exclusion of bacterial sepsis or worsened chronic medical condition.

Based on the proposed criteria for MIS-A (7), our patient presented within the proposed timeline of 12 weeks of recent COVID-19 infection. Nevertheless, our patient did not have MIS-A as he presented predominantly with respiratory complaints, later developed urosepsis, and lacked the required primary clinical criteria as highlighted in Table 2A. However, our patient fulfilled the laboratory criteria of a recent COVID-19 infection along with raised inflammatory markers and one of the secondary criteria; thrombocytopenia

Hemophagocytic lymphohistiocytosis is an uncommon condition characterized by immune dysfunction, persistent fever, and hemophagocytosis mediated by activated macrophages (26). HLH has been divided into primary and secondary subtypes. In both, there is immune dysregulation and cytokine storm leading to multiorgan dysfunction with fatal outcomes (27). Infection is the most common trigger for primary HLH, and malignancy is the most common cause in adults. Moreover, EBV is the most common infectious agent causing both primary and secondary HLH. (28, 29). Macrophage activation syndrome (MAS), a subtype of HLH, is characterized by excessive activation of macrophages leading to hyper-inflammation; and hyperferritinemia (30). Autoimmune and rheumatological conditions are associated with MAS (31).

There are case reports of SARS-CoV-2-induced sHLH diagnosed antemortem (11–15) and postmortem (1, 16–19) while the illness is/was ongoing, as shown in Table 2B. However, the literature is sparse about COVID-19 recovered cases developing sHLH (8–10), as shown in Table 2B. A study focusing on postmortem bone marrow biopsies of patients with COVID-19 showed histiocytic hyperplasia with hemophagocytosis (1). Elie Naous and colleagues have mentioned COVID-19-related HLH occuring 2 weeks after a documented clinical and biological recovery from COVID-19 illness in a 69-year-old woman (8). Our patient presented 4 weeks after severe COVID-19. He was initially suspected of having pirfenidone-induced liver injury with superadded lower respiratory tract infection, on a background of COVID-19 sequelae. Pirfenidone-related hepatotoxicity occurs with mild to moderate, asymptomatic serum transaminase elevations. The diagnosis of DILI is based on a high index of suspicion with the exclusion of other causes of liver dysfunction (32). Our patient was prescribed pirfenidone at another hospital as an off-label indication to prevent post-COVID-19 pulmonary fibrosis but this was stopped during the current hospitalization.

**Table 2B:** Tabulated comparison of studies on COVID-19-associated HLH.

**Cases Study**	**Year**	**No of cases**	**COVID status at diagnosis of HLH**	**HLH diagnostic** **tool used**	**H score**	**HLH 2004 criteria**	**Elevated sCD25**	**Reduced NK Cell activity**	**Bone marrow hemophagocytosis**	**Treatment**	**Outcome**
**Post COVID-19 cases**											
Naous et al. (8)	March 2021	1	Negative	HLH 2004		4	N/A	N/A	+	Dexamethasone+ Etoposide	Succumbed
Kalita P et al. (9)	August 2021	2	Negative	H-score	213/239	-	N/A	N/A	+	Dexamethasone	• Discharged • Not defined
Wiseman et al. (10)	September 2021	1	Negative	H score	197	3	N/A	N/A	+	Dexamethasone+ Etoposide	Initially improved then developed bronchopleural fistula and was on ECMO
Current study	2021	1	Negative	H score and HLH 2004	256	8	+	+	Hypoplastic marrow	Dexamethasone	Succumbed
**Ongoing COVID-19**											
**HLH-Diagnosed Antemortem**											
Verma et al. (11)	May 2021	1	Positive	HLH 2004	-	5	N/A	N/A	+	Dexamethasone	Succumbed
Schnaubelt et al. (12)	February 2021	3	Positive	H-score	239/197/256	-	N/A	N/A	N/A	• Methylprednisolone+IVIG • Methylprednisolone • Methylprednisolone+IVIG+ Anakinra	• Succumbed • Succumbed • Recovered
Thüsen et al. (13)	October 2020	1	Positive	H score; autopsy	180	-	+	N/A	+	N/A	Succumbed
Tholin et al. (14)	October 2020	1	Positive	HLH 2004	-	5	+	N/A	+	Tocilizumab IVIG	Recovered
Lima et al. (15)	July 2021	1	Positive	Bone marrow		3	N/A	N/A	+	IVIG	Succumbed
**HLH-diagnosed Postmortem**											
Retamozo et al. (16)	January 2021	60 **retrospective** **cases**	Variable	H score and HLH 2004	>169 (*n =* 7;11.6%)	5 (*n =* 8;13.3%)	N/A	N/A	83.3% (*n =* 15/18) +	Variable	46.6% expiry
Pérez et al. (1)	July 2020	3	Positive	HLH 2004	-	5	+	N/A	+	N/A	N/A
Torrón et al. (17)	February 2021	16	Positive	H score	>169 (*n =* 10)	-	N/A	N/A	+ (16/16)	Steroids (14) Tocilizumab (13)	Succumbed
Meng et al. (18)	April 2021	41 **retrospective** **cases**	Positive	H-score	>169 suspected HLH	-	N/A	N/A	N/A	Variable	100% died
Prilutskiy et al. (19)	2020	4	Positive	H score	1 had 217	-	N/A	N/A	BM-; Pulmonary lymph node+	Sarilumab, Anakinra	Succumbed

*BM, Bone Marrow; ECMO, extra-corporeal membranous oxygenation; IVIG, Intravenous Immunoglobulin; MPS, Methylprednisolone; N/A, Not Available*.

Hemophagocytic lymphohistiocytosis is a rare entity and a diagnosis of exclusion. In our case, after ruling out MIS-A and CSS, a diagnosis of HLH was contemplated because of raised inflammatory markers with persistent pancytopenia. We tried to eliminate most of the possible etiologies of sHLH and thus could attribute it to post COVID-19 infection. The probability of HLH was determined by the H-score given by Fardet et al.; our patient had a score of 256 points indicating a 99% probability of HLH (6). Previous case reports on post-COVID-19 HLH have relied mainly on H-score with scarce data on cytokine levels used in diagnosis for HLH (Table 2B) (8–10). The use of the H-Score for patients with COVID-19 has been questioned, and some authors recommend against using H-score. This is because of the low sensitivity of body temperature and the inability to distinguish between neutropenia and lymphocytopenia. Furthermore, there is a lack of published data on hypertriglyceridemia, splenomegaly, hepatomegaly, and marrow hemophagocytosis (16, 33). Most patients received steroids and had fatal outcomes despite treatment, as shown in Table 2B. Hence, we based our diagnosis of post-COVID-19 HLH on the HLH 2004 study criteria. This had a sensitivity of 91% and a specificity of 83.3% with a PPV of 97.6% and an NPV of 55.6% (34). The diagnosis of HLH needs any five out of the eight criterias: fever, splenomegaly, cytopenias affecting at least two of three lineages in the peripheral blood, hypertriglyceridemia (≥ 265 mg/dL) or hypofibrinogenemia (< 150 mg/dL), hemophagocytosis in bone marrow, spleen, or lymph nodes, low or absent NK-cell activity, hyperferritinemia (≥ 500 ng/mL), and raised sCD-25 levels (> 2SD) (26). Our patient met six of the eight criteria mentioned above. However, hemophagocytosis was not seen in the hypocellular bone marrow biopsy. Although hemophagocytosis is an essential component of HLH, it is not required for diagnosis. Bone marrow aspirates range from normocelluar to hypocellular to hypercellular in HLH. The prevalence of hemophagocytosis in patients with HLH ranges from 25 to 100% (35), which can also be caused by blood transfusions, infection, autoimmune disease, bone marrow failure, or red blood cell destruction (36–38). The presence of activated macrophages in the bone marrow or liver, as well as a comprehensive clinical examination, may help distinguish HLH from other causes of hemophagocytosis (39). As shown in Table 2B, most patients had hemophagocytosis, and 2 cases had no evidence of hemophagocytosis in the bone marrow. In our case, bone marrow was hypocellular for age and had < 15% cellularity. Autopsy evidence of HLH in patients with COVID-19 was first demonstrated by Prilutskiy et al. (Table 2B) (19).

In our case, MAS was ruled out after a negative autoimmune profile. Also, an extensive viral serology panel was negative; no apparent malignancy was detected on the CT scan or bone marrow biopsy and there was no blood transfusion. Hence, the clinical diagnosis of sHLH was attributed to COVID-19 based on the HLH-2004 criteria and raised H-score. Further, we considerd PACS in our patient as the patient presented to our hospital after 4 weeks of initial severe COVID-19 infection. Here, clinico-laboratory workup revealed sHLH after COVID-19 infection as a part of CSS. Admittedly, in this case report, considering sHLH as a delayed presentation of CSS due to COVID-19 or occurring as PACS is a debatable point.

There is also a possibility that sepsis could have triggered HLH in our patient. Although initial cultures were sterile, *E. coli* urosepsis was detected on the third day of admission, the very day we diagnosed him with HLH. A latent, persistent subclinical inflammatory state secondary to COVID-19 could have led to this presentation. Severe sepsis can present as the initial manifestation of HLH and hence, differentiating sepsis from HLH-sepsis overlap is quite challenging (40, 41). Clinical distinctions between severe sepsis and HLH might be ambiguous; prompt detection and management necessitate a high level of suspicion. More than 10% of patients with HLH die within 2 months of diagnosis due to bleeding in visceral organs, opportunistic infections due to neutropenia, or Multiple Organ Dysfunction Syndrome (MODS) (35). Our patient responded clinically and biochemically to dexamethasone and IVIG. However, due to immune dysfunction related to HLH, along with steroid-induced immunosuppression, he developed an opportunistic infection in the form of disseminated *C. auris* infection. As the initial fungal culture was sterile and the patient developed rashes on eleventh day, the temporality for causation of HLH by *C. auris* could not be established. *C. auris* has recently been linked to major outbreaks of invasive infections in healthcare facilities throughout the world (42). It is a multidrug-resistant pathogenic yeast that has high mortality rates (43). Globally, *C. auris* isolates are resistant to fluconazole in >90% of cases and amphotericin B in > 30% of cases (44). Our patient had *C. auris* on culture, resistant to fluconazole, but was sensitive to caspofungin. Despite treatment for HLH and candidemia, our patient developed ARDS, was intubated, and succumbed to the illness, thus highlighting the lethal sequence of events that challenged us in this case.

As this is a single case report, no consensus on guidelines and approach to management can be elucidated. More studies of such cases are needed to formulate strategies for the diagnosis and management of this fatal condition. Early detection of HLH among patients who have recovered from severe COVID-19 can be done by looking for the following pointers in patients (45): (1) ongoing remittent quotidian fever and being unresponsive to empiric antibiotic therapy; (2) hepatosplenomegaly; (3) falling ESR, leukopenia, and thrombocytopenia; (4) raised transaminases; (5) elevated CRP/ferritin/triglyceride; (6) hypofibrinogenemia.

Furthermore, with any of the above pointers, a panel of immunologic markers (elevated sCD25 and reduced NK cell cytotoxicity) should be ordered to detect HLH earlier, followed by guideline-directed treatment.

### Learning Points

Secondary HLH occurring in ongoing illness among patients of COVID-19 is an uncommon finding. We wish to highlight the occurrence of sHLH as a delayed presentation of CSS within the time frame of PACS.

• Post-COVID HLH is likely under-recognized, and mortality remains high, especially in adults; thus, prompt diagnosis and treatment are essential.
• Clinical and laboratory surveillance is necessary for a more extended period in patients with severe COVID-19.
• For diagnosis of sHLH among patients with COVID-19, using the HLH-2004 criteria for diagnosis seems more reliable than H-score.
• Indiscriminate use of immunosuppressive agents and pirfenidone among patients with severe COVID-19 may predispose them to fatal OIs since the patient is already in an immunosuppressed state due to steroids, COVID-19, and sHLH *per se*.

## Data Availability Statement

The original contributions presented in the study are included in the article/supplementary material, further inquiries can be directed to the corresponding author.

## Ethics Statement

Ethical review and approval was not required for the study on human participants in accordance with the local legislation and institutional requirements. The patients/participants provided their written informed consent to participate in this study.

## Author Contributions

All authors were involved in the management of the patient. SGau and GS collected all the clinically relevant data and completed the initial draft. SS and SGar had put their valuable input in editing and giving the shape to the final manuscript. All authors contributed to the article and approved the submitted version. Both SGau and SS made all the final changes and approved the author's proof.

## Conflict of Interest

The authors declare that the research was conducted in the absence of any commercial or financial relationships that could be construed as a potential conflict of interest.

## Publisher's Note

All claims expressed in this article are solely those of the authors and do not necessarily represent those of their affiliated organizations, or those of the publisher, the editors and the reviewers. Any product that may be evaluated in this article, or claim that may be made by its manufacturer, is not guaranteed or endorsed by the publisher.
